# An In-Depth Approach to the Associations between MicroRNAs and Viral Load in Patients with Chronic Hepatitis B—A Systematic Review and Meta-Analysis

**DOI:** 10.3390/ijms25158410

**Published:** 2024-08-01

**Authors:** Marina Manea, Ion Mărunțelu, Ileana Constantinescu

**Affiliations:** 1 Immunology and Transplant Immunology, University of Medicine and Pharmacy “Carol Davila”, 020021 Bucharest, Romania; 2 Centre of Immunogenetics and Virology, Fundeni Clinical Institute, 022328 Bucharest, Romania

**Keywords:** microRNA, hepatitis B virus, chronic disease, correlation

## Abstract

Scientists study the molecular activities of the hepatitis B virus (HBV). However, in vivo experiments are scarce. Some microRNAs are HBV-related, but their exact mechanisms are unknown. Our study provides an up-to-date view of the associations between microRNAs and HBV-DNA levels in chronically infected individuals. We conducted this large-scale research on five databases according to PRISMA guidance. Joanna Briggs Institute tools and Newcastle Ottawa Quality Assessment scores helped with quality evaluations. R 4.2.2 performed statistical computations for the meta-analysis. DIANA-microT 2023 and g:Profiler enriched the predictions of liver genes associated with miR-122 and miR-192-5p. From the 1313 records, we eliminated those irrelevant to our theme, non-article methodologies, non-English entries, and duplicates. We assessed associations between microRNAs and HBV-DNA levels. Overall, the pooled correlations favoured the general idea of the connection between non-coding molecules and viremia levels. MiR-122 and miR-192-5p were the most researched microRNAs, significantly associated with HBV-DNA levels. The connections between miR-122, miR-192-5p, let-7, miR-215, miR-320, and viral loads need further in vivo assessment. To conclude, this study evaluates systematically, for the first time, the correlations between non-coding molecules and viremia levels in patients. Our meta-analysis emphasizes potentially important pathways toward new inhibitors of the viral replication cycle.

## 1. Introduction

According to the European Centre for Disease Prevention and Control (ECDC), despite vaccination-driven politics, the infection with hepatitis B virus affects over 5.4 million people in Europe [1]. The Centre for Disease Control (CDC) expresses in its most recent guideline a great concern related to the diagnosis of asymptomatic chronically infected individuals [2]. A worldwide preoccupation is HBV screening [1,2] for rapid diagnosis and to avoid increases in transmission rates. Screening policies also promote disease awareness for the adequate treatment and evaluation strategy [2]. The World Health Organization (WHO) has implemented a series of screening tools for HBV, aiming at the elimination of disease transmission and the spread of severe complications [3]. Others have imagined volunteer-based strategies to increase awareness and improve HBV diagnosis [4]. However, the problem related to this illness remains unsolved, especially in the context of intense migration [5], drug usage in some communities [6], or gaps in providing cost-effective measures for HBV detection in other countries [7].

Chinese authors have envisioned a system based on viral load quantification to improve blood screening for HBV [8]. According to some researchers, the double-stranded form of HBV-DNA carries vital genetic information through the host’s serum [9]. Viral DNA loads change during the evolution of the disease, leading to the diagnosis of infection and helping in the patient’s evaluation [2]. HBV-DNA changes shape from a relaxed form into a circular nucleic acid inside the infected cell. Then, the viral DNA either gives the genetic pattern for new viral proteins or steps into an inactive phase inside the infected cell’s nucleus [9]. Some authors underline a connection between the remaining HBV-DNA in the infected cell and cancer formation [10]. Others link viremia to a prediction model for hepatocellular carcinoma (HCC) [11]. In a recent study, authors describe various molecular mechanisms triggered by a viral protein (HBx). These pathways cause methylation reactions on the circular form of HBV-DNA. As a result, the amount of viral nucleic acids increases. Part of the resulted double-stranded DNA remains in the host genome and may encourage, in time, HCC mechanisms [12]. According to some, serum HBV-DNA loads predict HCC recurrence after treatment [13]. In the occult HBV infection, HBV-DNA levels contribute to disease diagnosis because of the absence of serum viral proteins during this phase [9].

Therefore, studies incorporate the versatile role of HBV viral loads in pathogenesis. HBV-DNA is a deposit of genetic evidence, including the one related to mutations originating from treatment. Data acquisition concerning their evolution helps in improving therapeutic strategies [14]. Thus, the methods of HBV-DNA quantification are constantly improving, and some scientists describe new technologies for low-level viral DNA quantification [15].

However, there are knowledge gaps related to the behavior of HBV inside the infected cell. Therefore, contradictory opinions arise regarding the best prediction formula for complications like HCC [16]. The connections between viral mechanisms and molecular pathways lead to intense debate [17]. On the other hand, HBV-DNA is not the only important assay for a patient’s assessment [2]. Other viral proteins, such as HBx, play a central role in promoting a dangerous inflammatory environment and creating the premises for HCC [17]. Researchers seek for the exact molecular pathways that trigger HBV complications, as well as for the connection between HBV-DNA and other cellular elements. The knowledge gaps come from the complicated replication cycle of the virus interacting with the host’s immune system. Thus, researchers describe intricate mechanisms that do not fully explain the events and complications related to the chronic infection with HBV [18]. Some authors show that HBV-DNA stimulates immune responses and cGAS/STING pathways [19].

Scientists also depict microRNAs as key interactors between the virus and the infected host [20]. The synthesis of these non-coding molecules occurs in the cell’s nucleus, followed by their further structural changes in the cytoplasm. Mature microRNAs interact with the HBV polymerase, HBx protein, and other viral elements. They also trigger cellular and inflammatory events associated with HBV infection [21].

The variations of HBsAg in the patient’s serum seem to relate to the expressions of miR-301a-3p and miR-145-5p [18]. Other HBV proteins block miR-122 and trigger oncogenic pathways [17]. The associations between HBV and microRNAs in the development of HCC are under study [22]. Related to the response to viral treatments, studies included a series of microRNAs, such as miR-122, miR-192, and miR-320 [18]. MiR-21 and miR-29 are associated with the unknown increase in exosome levels in HBV [20]. Some scientists believe that miR-29 is related to the progression of HBV toward cirrhosis, but studies are scarce and inconclusive [21]. Other opinions claim that HBV can influence the expression of miR-141-3p and miR-425-5p to promote viral replication and invasion [17]. 

The biomarker quality of non-coding molecules in HBV infection needs further study, although some promising experiments were performed [18]. Scientists believe there is a connection between microRNAs and HBV-DNA, especially for miR-122 [21], but with unknown pathways. Assessing viral loads and microRNAs in patient samples can be complicated, and articles tend to include a limited, small number of individuals [23]. 

This meta-analysis is the first meticulous research on the association between microRNAs and HBV-DNA related to in vivo studies. The aim was to present an up-to-date situation in the context of human participants because most microRNA researchers chose the in vitro environment for their studies [18]. This meta-analysis is also the first study on a large pool of individuals from various articles conducted to assess correlations between microRNAs and HBV viral loads. We wanted to find a highly accurate and up-to-date answer related to the extent of the microRNA involvement in HBV replication. Therefore, a bioinformatics analysis explored potential connections between the microRNAs retrieved in this study. Our paper might improve new therapies based on the microRNA inhibition of the viral replication cycle [24].

## 2. Material and Methods

### 2.1. The Article Extraction Process

We developed a search strategy based on PRISMA guidelines [25]. The words used for article retrieval were first analyzed using Systematic Review Accelerator (https://sr-accelerator.com/, Bond University, Gold Coast, QLD, Australia; accessed on 29 June 2024) [26]. The final combination of elements from the search queries contained derivatives and abbreviations from “hepatitis”, “patient”, “hepatitis B virus”, “liver”, “viremia”, “HBV-DNA”, “miRNA”, and “microRNA”. We did not pose any search limits. The search process ended on 29 June 2024. We assessed all articles from inception till the last search date. The retrieval process also included five databases: PUBMED, Web of Science, Scopus, Cochrane Library, and Taylor and Francis.

### 2.2. The Selection Method

A thorough selection process of all articles was performed independently by two of the authors (M.M. and I.M.). We wanted to find calculated correlations between HBV-DNA and microRNAs. A pre-written form included the retrieved data. We also took into consideration some basic study details and detection techniques. Inclusion criteria followed the existence of English-written, full-text articles. Systematic Review Accelerator (https://sr-accelerator.com/, Bond University, Gold Coast, QLD, Australia; accessed on 29 June 2024) [26] helped with duplicate retrievals and exclusion. We did not include articles unrelated to the theme of our study. We also eliminated reviews, conference proceedings, letters to the editor, editorials, pre-prints, patent inquiries, guidelines, and retracted articles.

### 2.3. Data Acquisition and Quality Assessment

Extracted data were included in a pre-written form. The third author (I.C.) settled debates between the two screening authors. The final remaining articles provided information on in vivo studies related to chronically infected HBV patients. We included the associations between microRNAs and viral loads in a correlation meta-analysis. The main article archive was ZOTERO (http://www.zotero.org; accessed on 1 February 2023) [27]. The quality assessment tools came from two websites. Diagnostic articles were assessed using the quality tool of Joanna Briggs Institute (JBI- https://jbi.global/critical-appraisal-tools; accessed on 29 June 2024) [28]. This critical tool contains a series of “Yes” and “No” questions. Articles with more than 50% of “Yes” answers from the total available options were considered good quality research with moderate to low risk of bias. We evaluated the quality of cohort and case-control studies using the Newcastle Ottawa Quality Assessment Scale (NOS) [29]. A modified NOS scale [30] provided the quality evaluation method for cross-sectional articles. We analyzed only 7-star articles on NOS scales because these were classified as high-quality papers with a moderate to low risk of bias. The protocol of our meta-analysis is public in Supplementary Table S1. We did not register it. Supplementary Table S2 includes the PRISMA checklist of this paper (adapted after [31]).

### 2.4. A Bioinformatics Analysis on the Retrieved microRNAs

We performed a bioinformatics analysis on the most frequently found microRNAs in our meta-analysis. Therefore, DIANA-microT 2023 (DIANALab, University of Thessaly, Hellenic Pasteur Institute, Athens, Greece; https://dianalab.e-ce.uth.gr/microt_webserver/#/; accessed on 18 July 2024) [32] predicted a series of liver genes connected with serum miR-122 and miR-192-5p. We chose only miRBase’s high-confidence interactions with a 0.95 score threshold. Then g:Profiler (elixir Estonia, University of Tartu, Tartu, Estonia; https://biit.cs.ut.ee/gprofiler/page/contact; accessed on 18 July 2024) [33] enriched the retrieved liver genes. We considered significant a *p*-value under 0.01, adjusted with a Bonferroni correction.

### 2.5. Statistical Methods

Statistical evaluations and graphical representations were performed using R 4.2.2 software (R Foundation for Statistical Computing, Vienna, Austria) [34]. Cooper et al. [35] provided information for the meta-correlation analysis based on Fisher’s transformed correlations. We also performed a heterogeneity assessment based on Higgins et al. [36] formulae and *I*^2^ values (over 75% meant high inconsistency). Funnel plots, forest plots, and Egger’s test [37,38] evaluated data differences and bias. Subgroup analysis further evaluated data differences. Significance was related to *p*-values below 0.05. For the bioinformatics analysis we used a significant *p*-value under 0.01, adjusted by a Bonferroni correction for higher accuracy.

## 3. Results

### 3.1. The Article Selection Scheme

**Figure 1:**
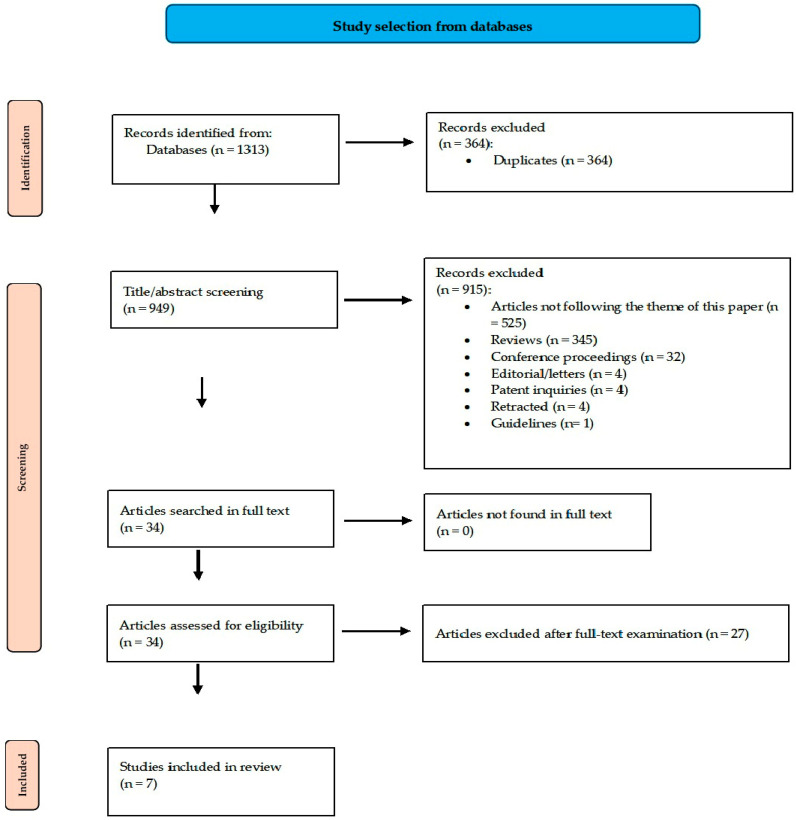
The selection scheme (adapted from the 2020 PRISMA statement [25]). Reasons for the exclusion of articles assessed for eligibility are depicted in Supplementary Table S3.

We retrieved 1313 articles (PUBMED—164, Web of Science—181, Cochrane Library—4, Scopus—182, and Taylor and Francis—782) and excluded 364 duplicates The second exclusion step included 915 records. Figure 1 depicts the reasons for their elimination. We discarded 525 articles because they did not follow the theme of our study. Non-article records were also discarded (345 reviews, 32 conference proceedings, 4 editorial/letters, and 4 patent inquiries). We also eliminated four retracted articles; 34 articles remained [39,40,41,42,43,44,45,46,47,48,49,50,51,52,53,54,55,56,57,58,59,60,61,62,63,64,65,66,67,68,69,70,71,72]. After full-text evaluation, we excluded 27 articles, mostly because their authors did not calculate correlation coefficients (see Supplementary Table S3 for further explanations). The final meta-correlation included seven articles. Figure 1 includes the search strategy’s details.

### 3.2. Main Article Data

We assessed seven articles with 541 chronic HBV patients. More than half of the studies were recent (written between 2021 and 2022) [69,70,71,72]. Most articles included Asian-origin participants [66,68,69,70,71,72]. We could find only observational studies with a cohort [67,68,70,72], and cross-sectional [66,69,71] methodology. Chronic hepatitis B (CHB) patients predominated [66,67,68,70,71,72]. Apart from one study [70], most articles had RT-PCR detections of microRNAs [66,67,68,69,71,72]. Most of the non-coding molecules were detected from patient serum samples [66,68,69,70,71,72]. Every study had a good and very good quality, according to NOS [29] and modified NOS [30] scores. Correlations were assessed with Pearson and Spearman tests. We were interested only in the calculated correlations between microRNAs and HBV-DNA loads. Therefore, we divided accordingly the results into two categories, and we performed separate interpretations (for Pearson and Spearman correlations). Table 1 shows these results.

### 3.3. Meta-Correlation of Spearman Results

**Figure 2:**
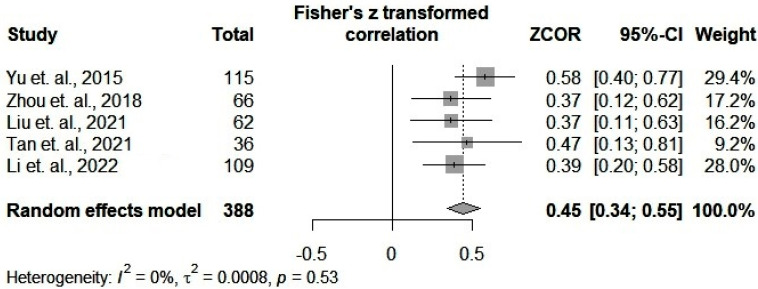
Forest plot of Spearman results (we included all the Spearman correlation coefficients between microRNAs and HBV-DNA levels assessed in the retrieved articles) [66,68,69,70,71].

We analyzed the Spearman correlations between microRNAs and viral loads. The pooled correlation was 0.45 (*p* < 0.0001), with 0% heterogeneity. However, this heterogeneity was uncertain (*p* = 0.53). Figure 2 depicts the forest plot of our results. We further assessed publication bias by examining the funnel plot (Supplementary Figure S1) and by performing the Egger’s test. We found no significant results. Next, a subgroup analysis assessed the potential causes of inconsistency (if any, considering the very low heterogeneity). For this, we divided studies into quality categories according to their NOS score. Articles with scores over 8 were considered very good. MicroRNA categories and patient sample types were the other two subgroups used. There was no significant difference between the quality scores of the articles. The pooled correlations between non-coding molecules and viral loads did not differ between microRNA subgroups (see Supplementary Figure S2). The difference between specimen samples did not cause significant heterogeneity. Because of the small number of studies, we could not perform further tests. Nevertheless, miR-122 proved to correlate with HBV-DNA (r = 0.38) without important inconsistencies.

### 3.4. Meta-Correlation of Pearson Results

**Figure 3:**
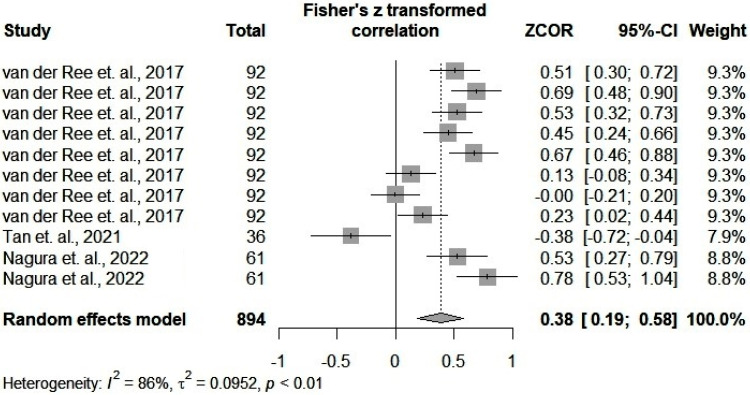
Forest plot of Pearson results (we included all the Pearson correlation coefficients between microRNAs and the HBV-DNA levels assessed in the retrieved articles) [67,70,72].

Pearson correlation analysis (Figure 3) showed a significant correlation between the studied microRNAs and HBV-DNA levels (*p* < 0.0001). Heterogeneity was also important (*I*^2^ = 86%, *p* < 0.01). The funnel plot and the Egger’s test showed no significant publication bias (Supplementary Figure S3). To evaluate heterogeneity, we used the same subgroups described previously. There was no significant difference between the quality scores of the articles. The difference between specimen samples did not cause significant heterogeneity. However, as we depicted in Supplementary Figure S4, we identified a significant difference between the correlations from various subgroups of microRNAs. In our meta-analysis, miR-192-5p correlated with HBV-DNA levels (r = 0.54). In this case, heterogeneity was low (*I*^2^ = 27%). We did not perform any further tests because of the small number of studies.

### 3.5. Bioinformatics Analysis

**Figure 4:**
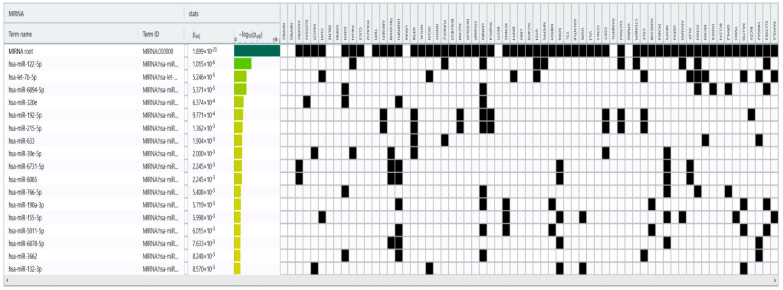
MicroRNAs associated with the liver genes retrieved from DIANA-microT 2023 [32]. The top row depicts the liver genes associated with miR-122 and miR-192-5p. The first column shows the predicted interactions between these genes and several microRNAs. The third column illustrates *p*-values adjusted after Bonferroni corrections.

**Figure 5:**
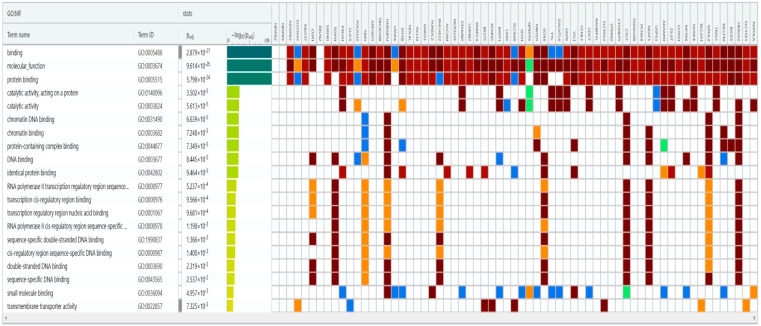
The molecular functions of the genes retrieved from DIANA-microT 2023 [32]. The top row depicts the liver genes associated with miR-122 and miR-192-5p. The first column shows the enriched molecular functions between these genes. The third column illustrates *p*-values adjusted after Bonferroni corrections.

In our meta-analysis, the most frequently found microRNAs were miR-192 and miR-122. miR-192-5p was studied on more patients than miR-192-3p. Therefore, we included miR-192-5p and miR-122 on a bioinformatics platform called DIANA-microT 2023 (DIANALab, University of Thessaly, Hellenic Pasteur Institute, Athens, Greece; https://dianalab.e-ce.uth.gr/microt_webserver/#/; accessed on 18 July 2024) [32]. We chose this software because of its recent updates and large interactions database. The results contained up-to-date information between the two retrieved microRNAs and liver genes. We took into consideration only high-confidence interactions between serum miR-192-5p, serum miR-122, and liver genes. We included the retrieved results on another bioinformatics platform called g:Profiler (elixir Estonia, University of Tartu, Tartu, Estonia; https://biit.cs.ut.ee/gprofiler/page/contact; accessed on 18 July 2024) [33]. The purpose was to obtain all the interactions between other microRNAs and the liver genes found on DIANA-microT 2023 (DIANALab, University of Thessaly, Hellenic Pasteur Institute, Athens, Greece; https://dianalab.e-ce.uth.gr/microt_webserver/#/; accessed on 18 July 2024) [32]. We considered significant only the high-confidence results, with *p*-values under 0.01, adjusted after a Bonferroni correction. Figure 4 and Figure 5 depict our findings. This bioinformatics analysis showed possible interactions between miR-122, miR-192-5p, and other microRNAs. They influenced transmembrane transport mechanisms and DNA transcription.

## 4. Discussion

Several cell culture experiments studied the connections between microRNAs and various HBV molecules. It was found that non-coding molecules probably interfere with inner signals connected with the spread of the virus inside the infected organism. The activity of microRNAs also relates to HBV replication. This is the case of miR-155, miR-122, miR-130a, and miR-501 [73]. On the other hand, researchers have shown in cell experiments that miR-192-5p interacts with HBV through autophagy signals and influences viral replication [74]. Scientists have also used bioinformatics to evaluate microRNA activity. They retrieved a link between miR-130a and the replication pathways [75]. Recent data show that HBV might promote its replication by inhibiting some microRNAs, such as miR-138-5p [76]. Other microRNAs have the potential to inhibit the progression of the viral cycle. Cellular experiments prove this hypothesis. Therefore, microRNAs are potential therapeutic alternatives for HBV infection [77]. MiR-122 [78] and miR-1236 [77] are potential future therapies.

Until now, the exact interactions between microRNAs and HBV replication remain undeciphered. The viral spread is preventable, provided the connections between non-coding molecules and HBV-DNA are known. New treatment options might relate to the microRNA activity in the cellular proliferation cycle [73]. In this meta-analysis, we aimed to assess, for the first time in a systematic manner, known data related to the associations between microRNAs and HBV-DNA. We selected only in vivo experiments because the goal was to explore the potential of real-life connections in HBV patients. The number of patients included was high, reaching 541 individuals. Overall, microRNAs seemed to correlate with viral loads independent of the technique used to assess their association (Spearman or Pearson test). However, we observed high heterogeneity in Pearson meta-correlation. The diverse behavior of each microRNA, otherwise already documented in several studies, might explain our findings [73,78,79]. For instance, studies have shown that miR-122 acts on genes such as ADAM17 (disintegrin and metalloprotease 17), CCNG1 (cyclin G1) [73], or pathways such as ADAR1 (adenosine deaminase acting on RNA-1) [78] and WNT/β-catenin (wingless-related integration site/beta-catenin) [79]. MiR-125b, another microRNA found in our meta-analysis, inhibits TP53 (tumor protein 53) and PI3K/AKT (phosphoinositide 3-kinase/mitogen-activated protein kinase) [79]. Therefore, miR-125b stimulates cellular proliferation [79], while miR-122 sometimes promotes and, in other circumstances, inhibits cellular expansion [73]. Consequently, a possible explanation for the difference between the Pearson correlations might arise from the diversity of modulating activities that microRNAs have.

Regardless of the method used for correlation assessment, microRNAs had different association patterns with HBV-DNA levels. Diverse molecular pathways that include complex interactions between non-coding molecules and proteins could explain our findings [80,81]. For instance, studies show the interaction between miR-125b, miR-210, and several genes or proteins involved in HCC formation [81]. On the other hand, miR-29 interferes with immune signals by inhibiting the production of IL-12 [80].

We did not observe differences in microRNA expression in various categories of specimen samples. However, recent studies contradict our findings [82]. A cause for these variations might be related to complex modulating activities that microRNAs exert on blood cells, especially on platelets [83]. However, we had few categories of specimen samples, so we could not accurately assess microRNA behavior.

Our meta-analysis also revealed that miR-122 and miR-192-5p correlated with HBV-DNA levels. However, the method of detection for the assessment of the non-coding molecular expression could have biased the degree of association. Research has shown that detection techniques can provide different results [84]. The influence of several confounders, such as the quantity of microRNA present in the specimen sample, can cause such differences [85]. There is no gold standard for the detection of non-coding molecules, so their expression is often differently interpreted [84]. MicroRNA variations related to the infection stage and the treatment [67,70] might have also biased our research.

In our study, miR-122 showed a moderate correlation with HBV viral loads (r = 0.38). However, various in vivo [86] and in vitro [87] studies have already documented the connection between miR-122 and HBV. This microRNA is more often associated with HCC formation [73,79,88,89], but it can also perform other modulatory effects (for instance in steatosis [73]). Other authors have also observed the correlation between miR-122 and viral loads [73]. Our high-quality systematic analysis tried to pool more accurate correlation coefficients between miR-122 and the levels of HBV-DNA. We managed this to some extent, considering the low heterogeneity of the retrieved studies. However, the small number of articles could have affected our research.

MiR-192-5p seemed to correlate with HBV-DNA levels to a greater extent than miR-122 (r = 0.54). Considering the low heterogeneity of this analysis, we could emphasize the association between miR-192-5p and viral loads. On the other hand, this microRNA is more often linked to metabolic disorders related to lipid intake [90] and glucose resistance [91]. In our meta-analysis, three studies contained miR-192-related associations [67,71,72]. Regardless of the statistics, all these articles presented moderate correlations between miR-192 and HBV-DNA. Despite Spearman or Pearson statistics, MiR-122 also showed significant associations with viral loads in various articles [67,69,70]. In our meta-analysis, the most studied microRNAs were MiR-122 and miR-192.

For a comprehensive view, we performed a bioinformatics analysis. We retrieved 65 liver genes connected to miR-122 and miR-192-5p. Further analysis found interactions between those genes and 15 other microRNAs. That led to several conclusions. First, miR-122, miR-192-5p, and several other microRNAs participated in complex pathways. Secondly, because of their connection with microRNAs correlated with HBV-DNA (miR-122 and miR-192-5p), some of the retrieved non-coding molecules could have influenced viral loads. However, this hypothesis needs further studies.

Studies depict the connection between some of the retrieved microRNAs and HBV. For instance, let-7b predicted early HBV-related HCC [92]. Some authors documented an interaction between HBx protein, miR-122, and let-7 [93]. We found one study depicting a moderate correlation between miR-320 and HBV-DNA [70]. The bioinformatics analysis linked miR-320e and miR-122 to the LMNB2 (lamin B2) gene (Figure 4). This gene is related to immune pathways in HCC [94]. Other studies link miR-320 with other molecular pathways in HBV-related HCC [95]. Therefore, the associations between miR-122 and the miR-320 family need further assessment in the HBV infection.

On the other hand, we found that miR-215-5p interacts with miR192-5p and influences KIF5B (kinesin family member 5B) genes. The kinesin proteins are known for their involvement in cellular transport through microtubules [96]. Some authors have shown that kinesins and microtubules enhance the release of exosomes [97]. Others suggest a connection between exosomal miR-192-5p, miR-215, and immune pathways in HBV [98]. The release of exosomes is the final part of the HBV replication cycle [9].

Our study had a series of limitations. First, we had no definitive conclusion because of the small number of articles for every microRNA. Second, we identified various sources of bias, unavoidable in current microRNA research. For instance, unresolved confounders arose from differences between molecular detection techniques. Potential heterogeneity between the selected patients could have influenced our meta-analysis. Furthermore, some individuals were treated [68,70]. Differences between such patients and those without treatment could have biased our results. The disease phase-related fluctuations in the expressions of microRNAs [73] potentially influenced our findings. Other limitations arose from our bioinformatics analysis. When predicting interactions, every software presumes that the connections happen simultaneously. However, real-life data contradicts this hypothesis [73].

Nevertheless, this study has strong assets. It is the first meta-analysis with a broad perspective on the correlations between microRNAs and HBV-DNA levels. Moreover, this study includes many patients. The high-quality methodology of our meta-analysis renders an accurate up-to-date presentation of the correlations between microRNAs and viral loads. Our study also brings new perspectives in molecular research. We illustrated which non-coding molecules were associated with viral loads in vivo. An in vitro setting might give a different, distorted impression of microRNA activity [99]. Moreover, bioinformatics emphasized several microRNAs potentially connected with HBV replication. Not least, our research illustrated the need for more in vivo studies about the activity of non-coding molecules. This might lead to the discovery of precise microRNA interactions with disease-specific elements.

## 5. Conclusions

This meta-analysis offers a high-quality, up-to-date view of the connections between certain microRNAs and HBV-DNA levels. Our study brings new future perspectives on the associations in the molecular environment. Researchers are already seeking new artificial inhibitors of the viral replicative cycle [100]. Therefore, our meta-analysis emphasizes some gaps in knowledge to be solved for finding a cure for HBV infection.

## Figures and Tables

**Table 1:** An overall view of each articles’ information.

Article Reference	Study Site	Article Type	Patient Counts	Patient Average Age	Identified microRNA	Detection Method	Correlation Assessment (between microRNAs and Viral Loads)	NOS Score *
Yu et al., 2015 [66]	China	Cross-sectional	CHB group: 115 HC: 20	CHB group: 52.84 ± 3.82 HC: 49.99 ± 5.16	miR-210 (serum samples)	RT-PCR	Spearman: r = 0.525 *p* < 0.001	7
van der Ree et al., 2017 [67]	Nether-lands	Cohort	CHB group: 92	Cohort 1: HBeAg positives- 35 ± 10 HBeAg negatives- 42 ± 12 Cohort 2: HBeAg positives- 35 ± 9 HBeAg negatives- 44 ± 10	miR-122-5p miR-125b-5p miR-192-5p miR-193b-3p miR-194-5p miR-200a-3p miR-204-5p miR-29a-5p (plasma samples)	RT-PCR	Pearson: miR-122-5p- r = 0.468 *p* = 0.01 miR-125b-5p- r = 0.599 *p* < 0.01 miR-192-5p- r = 0.483 *p* < 0.01 miR-193b-3p- r = 0.422 *p* < 0.01 miR-194-5p- r = 0.585 *p* < 0.01 miR-200a-3p- r = 0.130 *p* > 0.05 miR-204-5p- r = −0.003 *p* > 0.05 miR-29a-5p- r = 0.228 *p* < 0.05	8
Zhou et al., 2018 [68]	China	Cohort	CHB group HBeAg negative treated with NA: 66	CHB group: 31.55 ± 10	miR-125b (serum samples)	RT-PCR	Spearman: r = 0.353 *p* = 0.004 (baseline measurement)	9
Liu. et al., 2021 [69]	China	Cross-sectional	HBV group: 62 Controls: 11	HBV carrier group: 30.29 ± 11.19 CHB group: 38 ± 10.81 Cirrhosis group: 43.29 ± 6.28 Control group: 27.45 ± 6.2	miR-122 (serum samples)	RT-PCR	Spearman: r = 0.354 *p* = 0.005	7
Tan et al., 2021 [70]	China	Cohort	CHB group with HBeAg seroconversion after treatment: 36	CHB responsive group to treatment: 33.72 ± 10.47 CHB non-responsive group to treatment: 33.78 ± 8.72	miR-122-5p miR-320a-3p (serum samples)	Sequencing technique	Spearman: miR-122-5p- r = 0.438 *p* = 0.008 Pearson: miR-320a-3p- r = −0.366 *p* = 0.028	8
Li et al., 2022 [71]	China	Cross-sectional+ in vitro analysis	CHB group: 109 HC group: 20	-	miR-192-3p (serum samples)	RT-PCR	Spearman: r = 0.37 *p* = 0.0002	7
Nagura et al., 2022 [72]	Japan	Cohort	CHB group: 61	CHB group: 36.06 ± 8.35	miR-192-5p (serum samples)	RT-PCR	Pearson: baseline- r = 0.484 *p* < 0.001 Pearson: In week 24- r = 0.655 *p* < 0.001	9

* Data were illustrated as media ± standard deviation (SD). NOS—Newcastle Ottawa Quality Assessment, RT-PCR—real-time PCR, NA—nucleoside/nucleotide analogs, HBV—hepatitis B virus, CHB—chronic hepatitis B, HC—healthy control.

## Supplementary Materials

The following supporting information can be downloaded at: https://www.mdpi.com/article/10.3390/ijms25158410/s1.
