# Supplementary material for: An In-Depth Approach to the Associations between MicroRNAs and Viral Load in Patients with Chronic Hepatitis B—A Systematic Review and Meta-Analysis
Source: Int J Mol Sci. 2024 Aug 1;25(15):8410. doi: 10.3390/ijms25158410 (PMC11313658; doi:10.3390/ijms25158410)
Supplement: Supplementary file 1 [file ijms-25-08410-s001.zip › Figure S1.pdf]

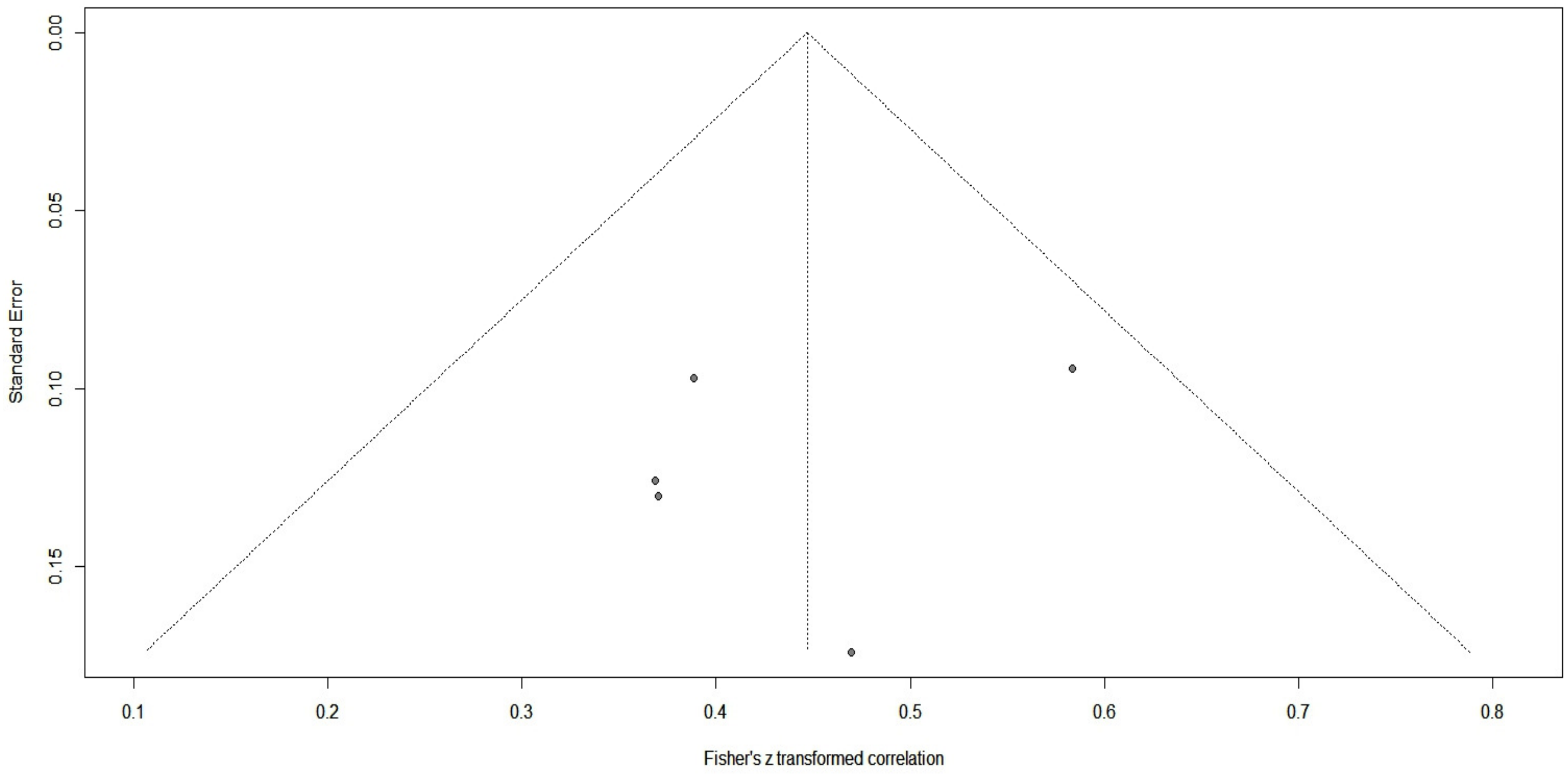

Figure S1. Funnel plot of Spearman results (we included all the Spearman correlation coefficients between microRNAs and the HBV-DNA level assessed in the retrieved articles)
