# Supplementary material for: An In-Depth Approach to the Associations between MicroRNAs and Viral Load in Patients with Chronic Hepatitis B—A Systematic Review and Meta-Analysis
Source: Int J Mol Sci. 2024 Aug 1;25(15):8410. doi: 10.3390/ijms25158410 (PMC11313658; doi:10.3390/ijms25158410)
Supplement: Supplementary file 1 [file ijms-25-08410-s001.zip › Figure S2.pdf]

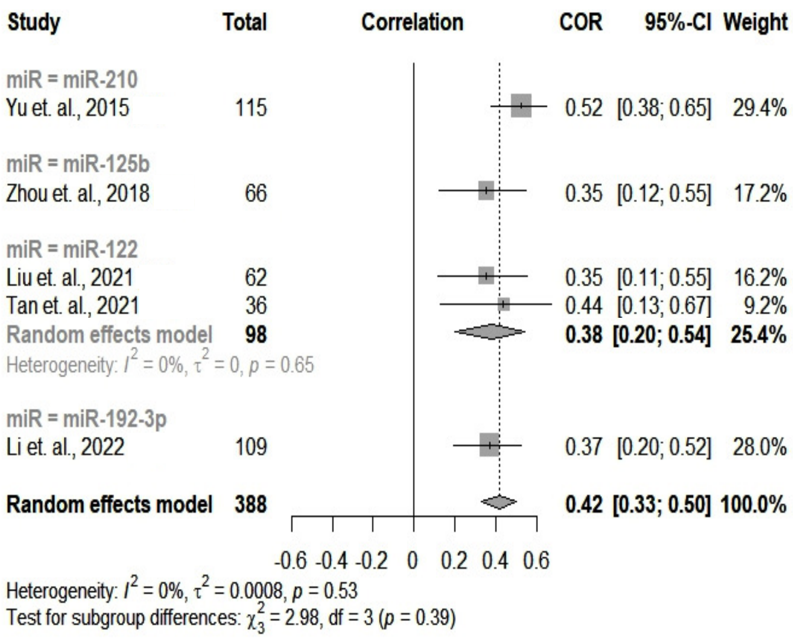

Figure S2: The difference between microRNA subgroups in Spearman correlations (all correlations were performed between microRNAs and viral loads) [66, 68-71].
