# Supplementary material for: An In-Depth Approach to the Associations between MicroRNAs and Viral Load in Patients with Chronic Hepatitis B—A Systematic Review and Meta-Analysis
Source: Int J Mol Sci. 2024 Aug 1;25(15):8410. doi: 10.3390/ijms25158410 (PMC11313658; doi:10.3390/ijms25158410)
Supplement: Supplementary file 1 [file ijms-25-08410-s001.zip › Supplementary Table S1.pdf]

Supplementary Table S1. The protocol of the meta-analysis

**Review questions**	What are the correlations between microRNAs and viral loads in chronic HBV patients?
**Searches**	The retrieval process included five databases: PUBMED, Web of Science, Scopus, Cochrane Library, and Taylor and Francis.
**Inclusion criteria**	Studies containing calculated correlations between microRNAs and HBV-DNA levels.<br>Articles written in English, available in full-text.
**Exclusion criteria**	Studies not having the inclusion criteria.<br>We also eliminated reviews, conference proceedings, letters to the editor, editorials, pre-prints, patent inquiries, guidelines, and retracted articles.
**Population, exposures, comparators, outcomes**	We included articles referring to calculated correlations between microRNAs and HBV-DNA levels.
**Data extraction**	Two authors (MM and IM) independently gathered, extracted and analysed data. All entries from inception until 29 June 2024 were added and analysed (see the process depicted in **Figure 1**). IC supervised the process and settled opinion differences. Software used for data extraction: Systematic Review Accelerator [26] and ZOTERO [27].
**Risk of bias and quality assessment**	Tools for diagnostic studies: Joanna Briggs Institute (JBI) [28]<br>Tools for cohort, case-control and cross-sectional studies: Newcastle-Ottawa Quality Assessment Scale (NOS) [29], modified NOS scale [30].
**Statistical analysis**	Software used: R 4.2.2 Software (R Foundation for Statistical Computing, Vienna, Austria) [34].<br>Method used: Fisher's z-transformed correlations<br>We also performed a heterogeneity assessment based on Higgins et al. [36] formulae and I^2 values (over 75% meant high inconsistency). Funnel plots, forest plots, and Egger's test [37-38] evaluated data differences and bias. Subgroup analysis further evaluated data differences. Significance was related to *p*-values below 0.05.

HBV-hepatitis B virus
