# Supplementary material for: An In-Depth Approach to the Associations between MicroRNAs and Viral Load in Patients with Chronic Hepatitis B—A Systematic Review and Meta-Analysis
Source: Int J Mol Sci. 2024 Aug 1;25(15):8410. doi: 10.3390/ijms25158410 (PMC11313658; doi:10.3390/ijms25158410)
Supplement: Supplementary file 1 [file ijms-25-08410-s001.zip › Supplementary Table S3.pdf]

Supplementary Table S3. Reasons for article exclusion after full-text assessment

Study	Reason for exclusion
Guo et al, 2011 [39]	Small number of patients with potential risk of bias- 25 individuals.
Waidman et al, 2012 [40]	Poor quality (NOS score= 5)
Arataki et al., 2013 [41]	Chinese article
Coppola et al., 2013 [42]	Correlation coefficient not calculated
Fu et al., 2013 [43]	Correlation coefficient not calculated
Winther et al., 2013 [44]	Correlation coefficient not calculated
Brunetto et al., 2014 [45]	Method inappropriate for our study-the authors calculate a miR-B index composed of various microRNAs, using elaborate mathematical computation- potential risk of high bias for our meta-correlation
Xie et al., 2014 [46]	Poor quality (according to JBI tool)
Xing et al., 2014 [47]	Poor quality (NOS score= 6)
Akamatsu et al., 2014 [48]	Correlation coefficient not calculated
Jin et al., 2015 [49]	Correlation coefficient not calculated
Mohamadkhani et al., 2015 [50]	Poor quality (NOS score= 5)
Xing et al, 2015 [51]	Poor quality (according to JBI tool)
Li et al., 2016 [52]	Correlation coefficient not calculated
Yu et al., 2016 [53]	Poor quality (NOS score= 6)
Zhou et al., 2016 [54]	HCC patients (with combined etiologies)
Qiao et al., 2017 [55]	Correlation coefficient not calculated

Yang et al., 2017 [56]	HBV-microRNA, not human microRNA
Akuta et al., 2018 [57]	Not in our theme (Potential acute HBV patients included)
Shen et al., 2018 [58]	HCC patients (with combined etiologies)
Li et al., 2019 [59]	Small number of patients with potential risk of bias- 14 individuals.
Yousefpouran et al., 2020 [60]	Small number of patients with potential risk of bias- 20 individuals.
Laleh et al., 2021 [61]	Poor quality (NOS score= 6)
Liu et al., 2021 [62]	Poor quality (NOS score= 5)
Gan et al., 2022 [63]	HBV-microRNA, not human microRNA
Loukachov et al., 2022 [64]	HBV-microRNA, not human microRNA
Loukachov et al., 2022 [65]	Poor quality (NOS score= 6)

HBV-hepatitis B virus; HCC-hepatocellular carcinoma; NOS-Newcastle Ottawa Quality Assessment Tool; JBI- Joanna Briggs Institute Quality Tool
